# The genomic structure of isolation across breed, country and strain for important South African and Australian sheep populations

**DOI:** 10.1186/s12864-021-08020-3

**Published:** 2022-01-04

**Authors:** Cornelius Nel, Phillip Gurman, Andrew Swan, Julius van der Werf, Margaretha Snyman, Kennedy Dzama, Klint Gore, Anna Scholtz, Schalk Cloete

**Affiliations:** 1 Department of Animal Sciences, Stellenbosch University, 7602 Stellenbosch, South Africa; 2 Animal Sciences, Western Cape Department of Agriculture, 7607 Elsenburg, South Africa; 3 Animal Genetics & Breeding Unit, University of New England, NSW 2351 Armidale, Australia; 4 School of Environmental and Rural Science, University of New England, 2351 Armidale, NSW Australia; 5 Department of Agriculture, Land Reform and Rural Development, Grootfontein Agricultural Development Institute, 5900 Middelburg, South Africa

**Keywords:** Across-country, Genetic variation, Merino, Population genetics, Sheep, SNP markers

## Abstract

**Background:**

South Africa and Australia shares multiple important sheep breeds. For some of these breeds, genomic breeding values are provided to breeders in Australia, but not yet in South Africa. Combining genomic resources could facilitate development for across country selection, but the influence of population structures could be important to the compatability of genomic data from varying origins. The genetic structure within and across breeds, countries and strains was evaluated in this study by population genomic parameters derived from SNP-marker data. Populations were first analysed by breed and country of origin and then by subpopulations of South African and Australian Merinos.

**Results:**

Mean estimated relatedness according to the genomic relationship matrix varied by breed (-0.11 to 0.16) and bloodline (-0.08 to 0.06) groups and depended on co-ancestry as well as recent genetic links. Measures of divergence across bloodlines (F_ST_: 0.04–0.12) were sometimes more distant than across some breeds (F_ST_: 0.05–0.24), but the divergence of common breeds from their across-country equivalents was weak (F_ST_: 0.01–0.04). According to mean relatedness, F_ST_, PCA and Admixture, the Australian Ultrafine line was better connected to the SA Cradock Fine Wool flock than with other AUS bloodlines. Levels of linkage disequilibrium (LD) between adjacent markers was generally low, but also varied across breeds (r^2^: 0.14–0.22) as well as bloodlines (r^2^: 0.15–0.19). Patterns of LD decay was also unique to breeds, but bloodlines differed only at the absolute level. Estimates of effective population size (N_e_) showed genetic diversity to be high for the majority of breeds (N_e_: 128–418) but also for bloodlines (N_e_: 137–369).

**Conclusions:**

This study reinforced the genetic complexity and diversity of important sheep breeds, especially the Merino breed. The results also showed that implications of isolation can be highly variable and extended beyond breed structures. However, knowledge of useful links across these population substructures allows for a fine-tuned approach in the combination of genomic resources. Isolation across country rarely proved restricting compared to other structures considered. Consequently, research into the accuracy of across-country genomic prediction is recommended.

**Supplementary Information:**

The online version contains supplementary material available at 10.1186/s12864-021-08020-3.

## Background

In South Africa (SA) and Australia (AUS), sheep production is dominated by a few major breeds. In SA, wool- and dual-purpose breeds such as the Merino, Dohne Merino and the SA Mutton Merino (SAMM) contributed nearly 70 % of all weaning weight records submitted to the National Small Stock Improvement Scheme between 2010 and 2011 [1]. In AUS, Australian Sheep Breeding Values are predominantly defined by three major branches as Merino, maternal (majority Border Leicester and Coopworth) and terminal (majority Poll Dorset and White Suffolk) breed groups [2]. Several breeds are in common between the SA and AUS sheep industries. The AUS and SA Merino share a distant relationship from the original Merino thought to have originated from Spain around the 1700s [3] and the AUS Merino was originally a composite of European, Asian and African strains [4]. Besides this ancestral relationship, a SA Merino resource flock has had intermittent genetic links to the AUS Merino by using sires originating from AUS [5], but this is the only known genetic exchange in recent years. Additional breeds in common include the South African Meat Merino (SAMM) pure breed and composites like the Dohne Merino and the Dorper meat breeds [5] which originated in South Africa and were exported to Australia.

The use of genomic information to increase the accuracy of Australian Sheep Breeding Values has been well established [2], but is still to be developed for South African sheep [6]. By establishing well-recorded reference populations, genomic tools could facilitate the inclusion of hard-to-measure traits by predicting the genetic merit of animals without phenotypes. This is of specific interest in the challenging environment underlying SA sheep production [1]. With breeds in common across countries, there could be potential to facilitate further development by combining genomic resources, but this would hinge on the compatibility of genomic pools of varying origin.

Population genetic parameters such as linkage disequilibrium (LD) and effective population size (N_e_) describe the diversity of populations and are important factors in the accuracy of genomic selection [7]. The more diverse populations are, the higher the sample size required to reach set levels of accuracy [8]. The level of LD, in turn, can be directly related to the level of inbreeding [9] and studies have emphasized the positive effect of relatedness between candidate and reference populations [10, 11]. Livestock breed formation is associated with restricted gene flow and different breeds are generally connected by weak, distant relationships. Results from multi-breed reference populations [12] suggest that additional genotypes might not be beneficial, or could even be detrimental if they originate from a distant group. This is of interest currently, since the implications of isolation ‘within breed’ - such as separation across country, strain, or flock - could be analogous to the ‘across breed’ situation, depending on the extent to which gene flow is restricted. However, the complicated genetic background of sheep [13] implies that genetic connectedness could be highly variable and rely on the unique population histories of each case of isolation between two groups.

Given the influential role of the Merino breed in both AUS and SA, a combined reference population for Merino sheep is of specific interest, but requires consideration of population structure. This breed has been characterised by a diverse genetic foundation using marker data [13–16]. Specifically, the AUS Merino consists of strong substructures, termed ‘lines’ or ‘strains’, originally classified partially by performance in key economic traits, including body weight, fleece weight, and wool fibre diameter [17]. Historically, they were separated by production environments and selection objectives, but the relatively recent implementation of technologies like artificial insemination has facilitated across population genetic links [18]. Genetic variation across these AUS Merino lines derived by both quantitative analysis [18] and genomic data [17, 19, 20] suggested an important role of these lines/strains in the genetic architecture of the AUS Merino. In turn, South Africa hosts multiple Merino resource flocks with widespread recording for hard-to-measure traits [1, 5] that could be a valuable contribution to a reference population enhancing prediction for SA industry animals. However, these flocks tend to be managed as selection experiments with a low genetic exchange with the wider industry. Also, since only selected flocks have recent links to specific bloodlines within the AUS Merino, the across-country relationships would be variable across subpopulations within SA and AUS Merinos.

The SA and AUS sheep populations are thus important examples of segregated genetic groups with varying histories that determine their linkage. While exact breeding histories are not known, modern genotyping platforms that make use of Single Nucleotide Polymorphisms (SNP) allow for informative estimates of genetic variation and relatedness across and within population groups, including examples related outside of pedigrees.

The objective of this study was to quantify a range of population genomic parameters from a medium density (50 K) SNP data panels to characterize genetic variation between and within subpopulations defined by different hierarchical structures such as breed, country, bloodline or flock of origin. The results should contribute to the knowledge of the genetic make-up underlying these sheep populations and could also facilitate the combining of genomic resources between AUS and SA, with a particular interest in building toward across country genomic prediction of Merinos.

## Results

### Heterozygosity and inbreeding

The average expected (${H}_{exp}$) and observed (${H}_{obs}$) levels of heterozygosity were moderately variable across breed groups, but across-country differences of common breeds were small (Table 1). ${H}_{exp}$ ranged from 0.33 for the Border Leicester to 0.38 for the AUS Merino. ${H}_{obs}$ ranged between 0.32 for the Border Leicester and 0.37 for the Coopworth, Corriedale and White Suffolk. Across Merino bloodlines, ${H}_{exp}$ ranged between 0.34 for Fine-Medium-2 to 0.37 for the Ultrafine and Grootfontein lines, a narrower range than that across breed groups. Bloodlines displayed similar ${H}_{obs}$ with all means between 0.36 and 0.37.

**Table 1:** Marker *h*eterozygosity (H_exp_ and H_obs_) (± SD) and *g*enomic *i*nbreeding (F_VR_ ± SD) for breed and Merino bloodline groups for respective countries

**Population**					**F**_**VR**_		
*Breed*	*Country*	***N***	**H**_**exp**_** (± SD)**	**H**_**obs**_** (± SD)**	***Min***	***Mean (± SD)***	***Max***
**Merino**	**AUS**	918	0.38 ± 0.12	0.36 ± 0.12	-0.15	0.09 ± 0.04	0.25
**Dohne Merino**		30	0.36 ± 0.14	0.36 ± 0.16	0.06	0.11 ± 0.03	0.20
**Dorper**		276	0.35 ± 0.14	0.35 ± 0.14	0.10	0.17 ± 0.04	0.30
**SAMM**		14	0.36 ± 0.14	0.36 ± 0.17	0.02	0.17 ± 0.06	0.28
**Border Leicester**		542	0.33 ± 0.15	0.32 ± 0.15	0.14	0.20 ± 0.03	0.32
**Coopworth**		114	0.35 ± 0.14	0.37 ± 0.15	0.07	0.13 ± 0.04	0.26
**Corriedale**		26	0.37 ± 0.13	0.37 ± 0.15	0.06	0.11 ± 0.03	0.18
**Poll Dorset**		400	0.34 ± 0.15	0.35 ± 0.15	0.10	0.19 ± 0.03	0.31
**White Suffolk**		247	0.37 ± 0.13	0.37 ± 0.14	0.02	0.12 ± 0.04	0.32
**Merino**	**SA**	697	0.37 ± 0.13	0.36 ± 0.13	0.00	0.09 ± 0.04	0.26
**Dohne Merino**		60	0.37 ± 0.13	0.37 ± 0.14	0.06	0.09 ± 0.02	0.14
**Dorper**		86	0.35 ± 0.14	0.34 ± 0.14	0.15	0.21 ± 0.03	0.31
**SAMM**		57	0.35 ± 0.14	0.35 ± 0.15	0.13	0.18 ± 0.03	0.29
**Dormer**		42	0.34 ± 0.15	0.34 ± 0.16	0.15	0.21 ± 0.03	0.27
*Bloodline*	*Country*						
**Ultrafine**	**AUS**	270	0.37 ± 0.13	0.37 ± 0.13	-0.17	0.08 ± 0.03	0.19
**Fine-Medium-1**		224	0.35 ± 0.14	0.37 ± 0.15	-0.17	0.07 ± 0.04	0.24
**Fine-Medium-2**		133	0.34 ± 0.15	0.37 ± 0.17	-0.01	0.05 ± 0.05	0.22
**Strong**		291	0.35 ± 0.14	0.36 ± 0.15	-0.16	0.07 ± 0.03	0.17
**Elsenburg**	**SA**	400	0.36 ± 0.14	0.36 ± 0.14	-0.04	0.03 ± 0.04	0.16
**Langgewens**		14	0.36 ± 0.13	0.39 ± 0.18	-0.03	0.02 ± 0.05	0.15
**Grootfontein**		115	0.37 ± 0.13	0.37 ± 0.14	-0.01	0.06 ± 0.04	0.22
**Industry**		41	0.36 ± 0.14	0.36 ± 0.15	0.00	0.07 ± 0.04	0.16
**Cradock**		127	0.36 ± 0.13	0.37 ± 0.14	0.02	0.06 ± 0.02	0.14

The mean level of inbreeding (F_VR_; derived from the diagonal of $\boldsymbol{G}$) varied considerably across breed groups (Table 1). The F_VR_ of AUS and SA Merinos and SA Dohne Merino was lowest (0.09). In turn, the SA Dorper and SA Dormer displayed the highest F_VR_ (0.21), while F_VR_-values of the AUS Border Leicester (0.20) and Poll Dorset (0.19) were also high. A narrower range (0.02 to 0.08) of generally low F_VR_ (derived from the diagonal of ${\boldsymbol{G}}_{\boldsymbol{M}}$) values was observed across the Merino bloodlines (Table 1). However, most groups consisted of a wide range of individual F_VR_ estimates indicating that animals lowly inbred (or outbred) and highly inbred is common in most populations.

### Pairwise F_ST_ statistics

The F_ST_ measure of population divergence also varied considerably across pairwise comparisons of breed groups (Table 2). The genetic distance of breed groups to their across-country counterparts was small with F_ST_ values of 0.01, 0.02, 0.03 and 0.04 for the Dorper, SAMM, Dohne Merino and Merino, respectively. Across breeds, low F_ST_ values were observed between Merino and Dohne Merino breeds in both SA and AUS (0.05–0.06). With consistently high F_ST_ estimates, the Border Leicester and Poll Dorset breeds diverged highly from all SA populations reported here, but a similar pattern was observed in relation to AUS breed groups (Table 2). The Coopworth and White Suffolk breeds were also distant across most comparisons, but to a lesser extent.

**Table 2:** Genetic divergence as pairwise F_STt_ estimates between breed groups across and within Australia and South Africa

*Across Country*		**M**	**DM**	**DR**	**SAMM**	**BL**	**COO**	**COR**	**WS**	**PD**
		**AUS**								
**Merino**	**SA**	0.04	0.07	0.13	0.09	0.19	0.13	0.08	0.11	0.17
**Dohne Merino**		0.05	0.03	0.13	0.07	0.2	0.13	0.08	0.11	0.18
**Dorper**		0.11	0.14	0.01	0.15	0.23	0.16	0.12	0.12	0.16
**SAMM**		0.08	0.09	0.16	0.02	0.23	0.16	0.11	0.13	0.2
*Within Country*										
**Merino**	**AUS**		0.06	0.11	0.08	0.17	0.12	0.06	0.09	0.15
**Dohne Merino**			-	0.15	0.09	0.22	0.15	0.09	0.12	0.19
**Dorper**			-	-	0.16	0.23	0.17	0.13	0.12	0.16
**SAMM**			-	-	-	0.23	0.16	0.1	0.13	0.2
**Border Leicester**			-	-	-	-	0.12	0.14	0.18	0.24
**Coopworth**			-	-	-	-	-	0.09	0.12	0.19
**Corriedale**			-	-	-	-	-	-	0.09	0.16
**White Suffolk**			-	-	-	-	-	-	-	0.11
		**SA**								
**Merino**	**SA**		0.05	0.12	0.10					
**Dohne Merino**		-	0.13	0.07						
**Dorper**		-	-	0.15						

Pairwise F_ST_ values (Table 3) showed that the Merino bloodlines were defined by a strong structure. However, evidence of a pattern of higher divergence across bloodlines from different countries was weak, such that the Strong and both ‘Fine-Medium’ lines were as diverged from their AUS contemporaries as from the SA groups. The largest F_ST_ distance was between the Strong and Fine-Medium-2 lines (0.12) and the smallest between the Industry and Grootfontein lines (0.04). All the comparisons within SA-only bloodlines were below 0.08. Of note was the low across-country estimate observed between the Cradock and Ultrafine lines (0.05). Also, most of the within-country AUS relationships between bloodlines were between 0.08 and 0.11, a magnitude similar to some F_ST_ values observed across breeds (Table 2).

**Table 3:** Genetic divergence as pairwise F_ST_ estimates between Merino bloodline groups

	Grt	Industry	Cradock	Ultrafine	FM-1	FM-2	Strong
**Elsenburg**	0.06	0.06	0.08	0.07	0.10	0.11	0.11
**Grootfontein**	-	0.04	0.05	0.06	0.09	0.10	0.10
**Industry**	-	-	0.06	0.06	0.09	0.10	0.10
**Cradock**	-	-	-	0.05	0.09	0.10	0.11
**Ultrafine**	-	-	-	-	0.08	0.09	0.09
**Fine-Medium-1**	-	-	-	-	-	0.11	0.10
**Fine-Medium-2**	-	-	-	-	-	-	0.12

### Genomic relatedness

**Fig 1:**
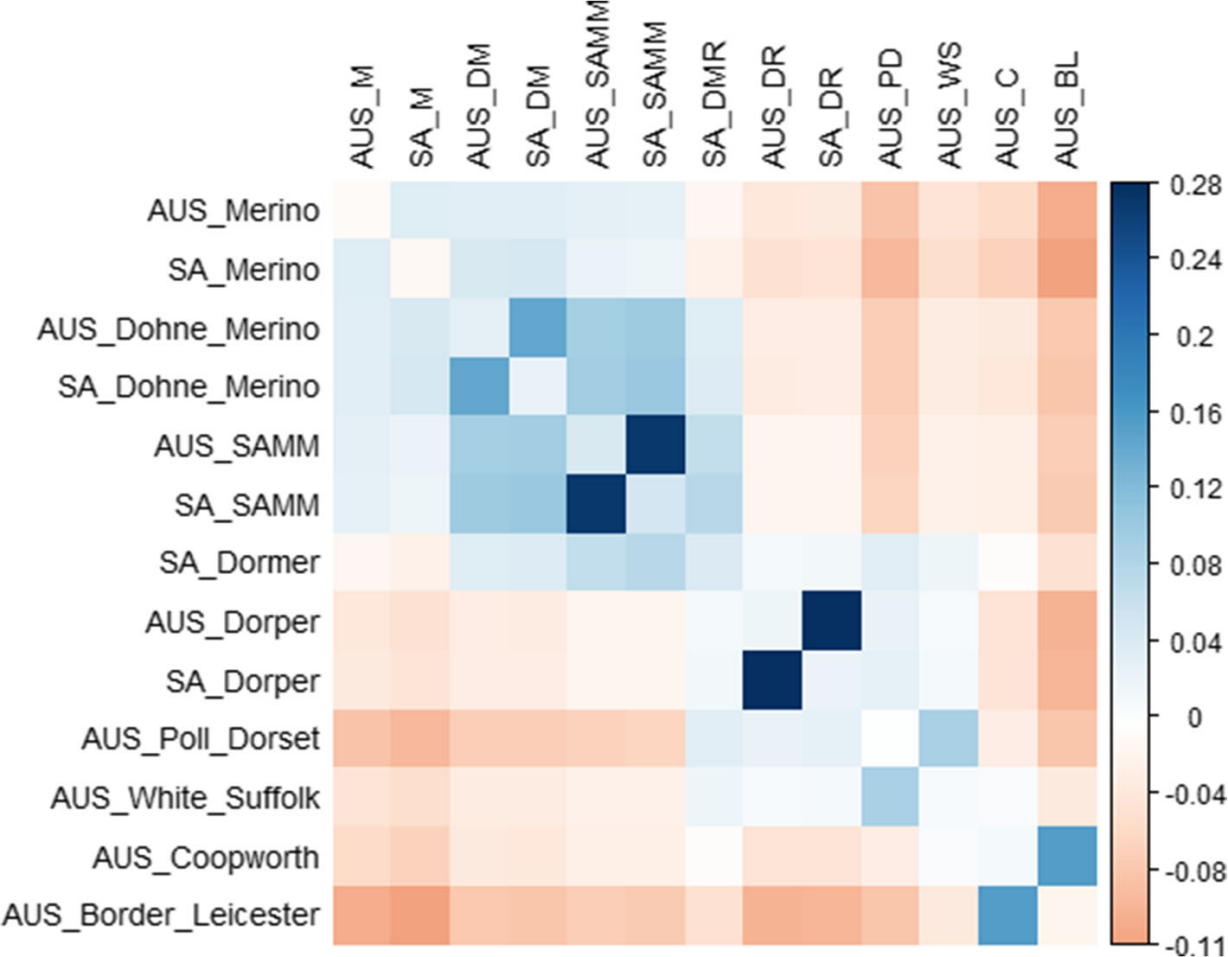
Pairwise estimates of mean relatedness of breed groups according to ***G***. The diagonal of the Fig. was replaced by a vector containing the mean relatedness to all other groups. Column abbreviations are a concatenation of country and breed as M: Merino, DM: Dohne Merino, DMR: Dormer, DR: Dorper, PD: Poll Dorset, WS: White Suffolk, C: Coopworth, BL: Border Leicester

Internal and across population relatedness was only reported for a subset of breed groups. Preference was given to include Merino and Merino types, breeds in common across countries and groups that represent the other major branches of AUS terminal and maternal types. Groups varied considerably in their levels of internal relatedness, determined by the mean (excluding diagonal) genetic relationship of animals to their contemporaries in the same breed or bloodline group (Table S1). The AUS Merinos, on average, were lowly related (0.09), which was in strong contrast to the Border Leicester group (0.44). In the mean relatedness across groups, most relationships ranged around 0. Exceptions were the cross-country comparisons for the Dohne Merino, SAMM and Dorper breeds where mean levels of 0.14, 0.27 and 0.28 were observed, the latter two results being particularly high. Measures involving the Border Leicester made up both the highest (0.16, Coopworth) and lowest (-0.11, SA Merino) result across different breeds. Discernable structures, or across breed ‘families’ were present within the relationships across breeds (Fig. 1). A lowly positive block separated Merino and Merino-like breeds, but the close relationships between some dual-purpose breeds were particularly apparent. The SA Dormer terminal sire breed bridged the Merino related groups to the pure meat-type Dorper, which grouped with the Poll Dorset and White Suffolk.

**Fig 2:**
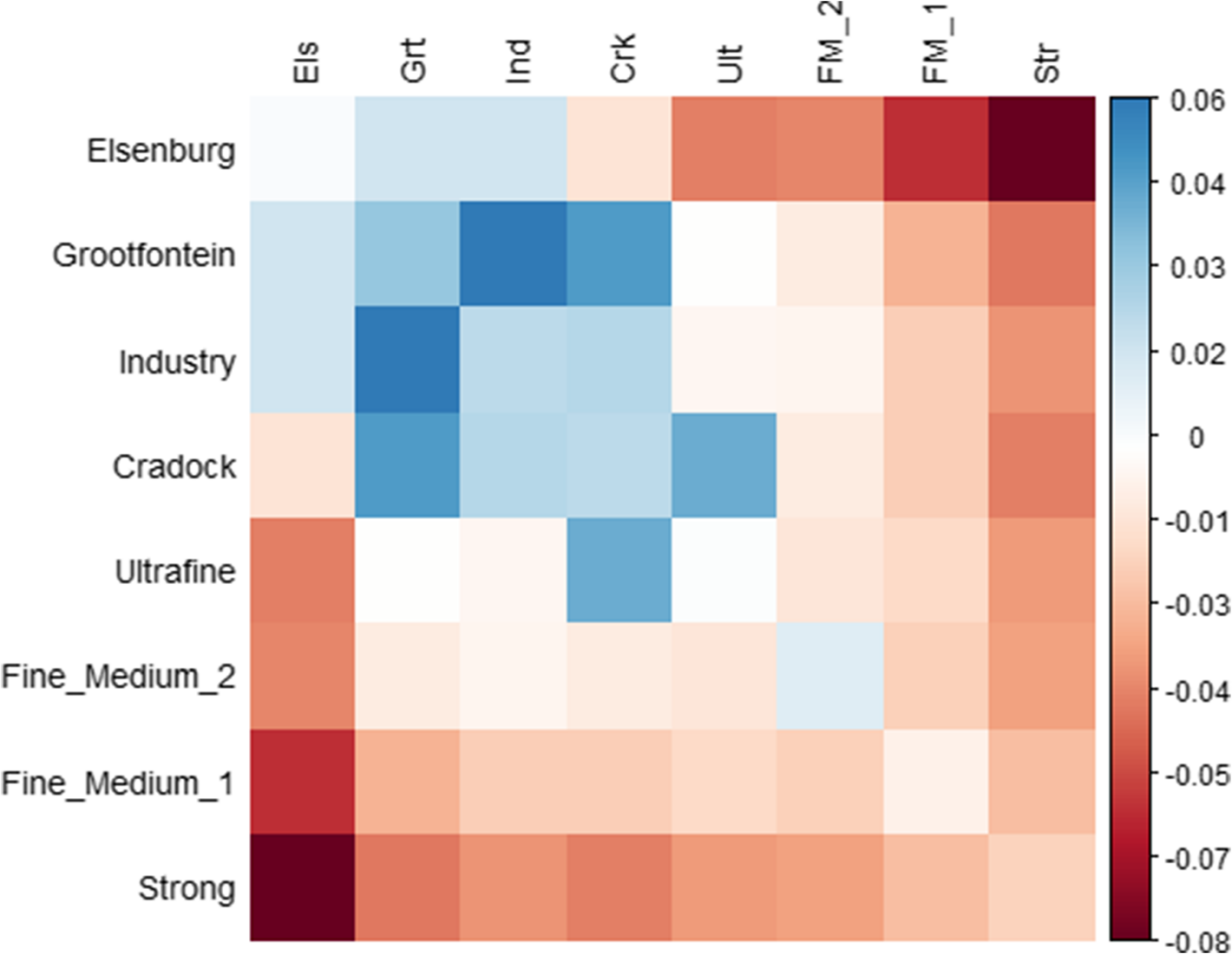
Pairwise estimates of mean relatedness of Merino bloodline groups according to ${\boldsymbol{G}}_{\boldsymbol{M}}$. The diagonal of the Fig. was replaced by a vector containing the mean relatedness to all other groups. Column abbreviations of bloodlines are Els: Elsenburg, Grt: Grootfontein, Ind: Industry, Crk: Cradock, Ult: Ultrafine, FM_2: Fine_Medium_2, FM_1: Fine_Medium_1, Str: Strong

The range of internal relatedness of Merino bloodline groups was smaller than for breed groups, but there were notable differences amongst them (Table S2). According to ${\boldsymbol{G}}_{M}$, animals in the Ultrafine (0.10) and the SA lines (0.11 to 0.14) were lowly related within their populations (Table S2). The Fine-Medium-2 line had the highest internal relatedness at 0.25, while high values of 0.18 and 0.20 were also observed for Fine-Medium-1 and the Strong line, respectively. The mean relationships across bloodlines in ${\boldsymbol{G}}_{M}$ are presented in Fig. 2. The only positive relationships are amongst SA groups with the notable exception of a positive relationship between the AUS Ultrafine and SA Cradock lines. Notably, this across-country relationship is the only positive estimate observed for the Ultrafine group. The remaining relationships, as well as the comparisons among AUS lines in general, were all lowly negative.

### Principal component analysis and admixture

**Fig 3:**
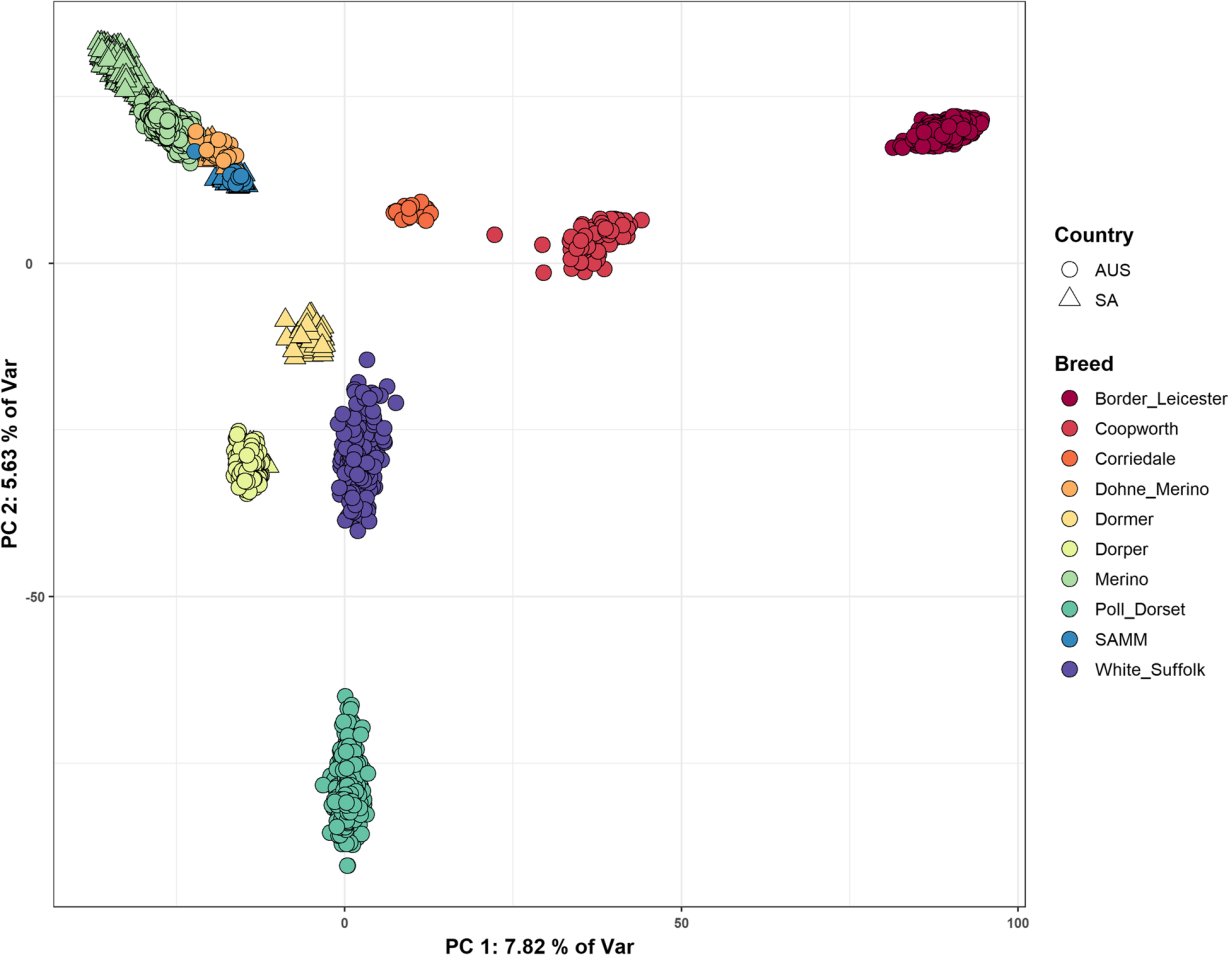
the first (PC1) and second (PC2) principal components of animals identified by breed and country of origin

A decomposition of the genomic relationship matrix (GRM) into its initial principal components provided a clear distinction between animals by breed structure (Fig. 3). Plotting against the first (PC1) and second (PC2) principal components separated all breeds except the Merino and Merino-like breeds, with no discernable separation by country for any breed groups in common between SA and AUS. In both PC1 and PC2 the Merino, Border Leicester and Poll Dorset were the most distant breeds. PC1 separated the Merino and Poll Dorset from the Border Leicester group, and PC2 separated the Merino, Corriedale and Border Leicester groups, from the remaining meat breeds with the largest distance observed between the Merino and Poll Dorset group. The proportion of variation (POV) explained by PC1 and PC2 (7.82 % and 5.63 %, respectively) is small, and the cumulative POV explained by principal components 1 to 40 showed an asymptotic trend at a low percentage (Fig. S5a). The first 20 principal components accounted for roughly 28 % and the first 40 for 32 % of the total observed variation. In succeeding principal components (PC3 to PC14; Fig. S2), breeds generally remained in group clusters, with the exception of Merino groups. However, there was little indication of across-country groups separated by different clusters within these components.

**Fig 4:**
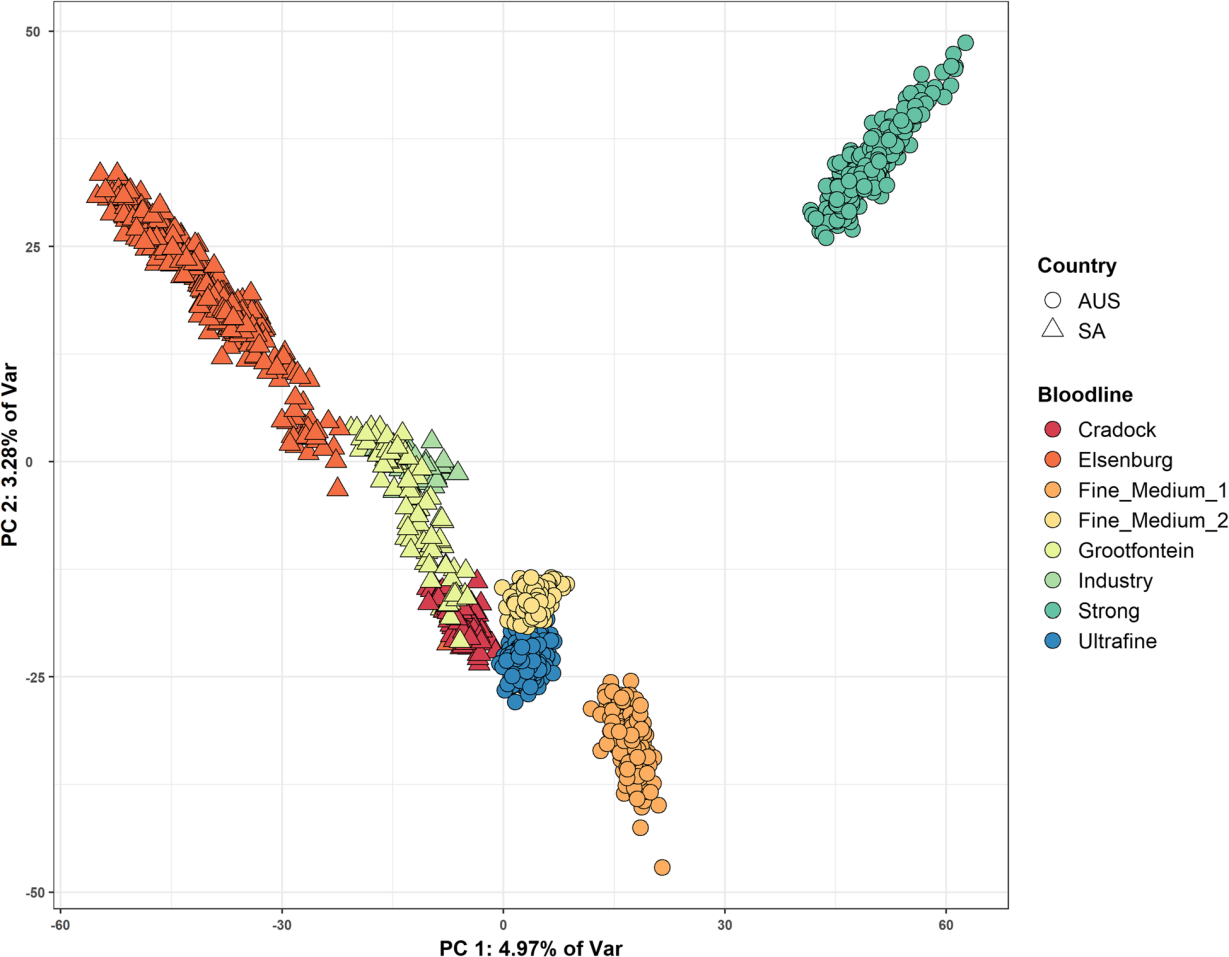
the first (PC1) and second (PC2) principal components of Merinos identified by bloodline and country of origin

PCA of the Merino bloodlines (Fig. 4) showed less discrete clustering compared to the breed groups (Fig. 3) but were still clearly discernable across PC1 and PC2. PC1 separated bloodline populations by country of origin, but to varying degrees.

All animals with positive values of PC1 were from AUS, whereas those with negative values of PC1 were all linked to SA. In PC1, the Elsenburg and Strong lines were separated the most. The Cradock line clustered near the Ultrafine and Fine-Medium-2 lines. PC2 separated the Elsenburg line and Strong lines from the Fine-Medium-1 line and an overlap between the Cradock and Fine-Medium-2 and the Ultrafine lines was observed. In general, the lines of AUS origin had more distinct clusters compared to the SA lines which varied more within groups on PC1 and PC2. The POVs explained by PC1 and PC2 (4.97 % and 3.28 %, respectively; Fig. S5a) of Fig. 4 were smaller than those observed by PC1 and PC2 that separated breeds (Fig. 3) but not by a large margin.

**Fig 5:**
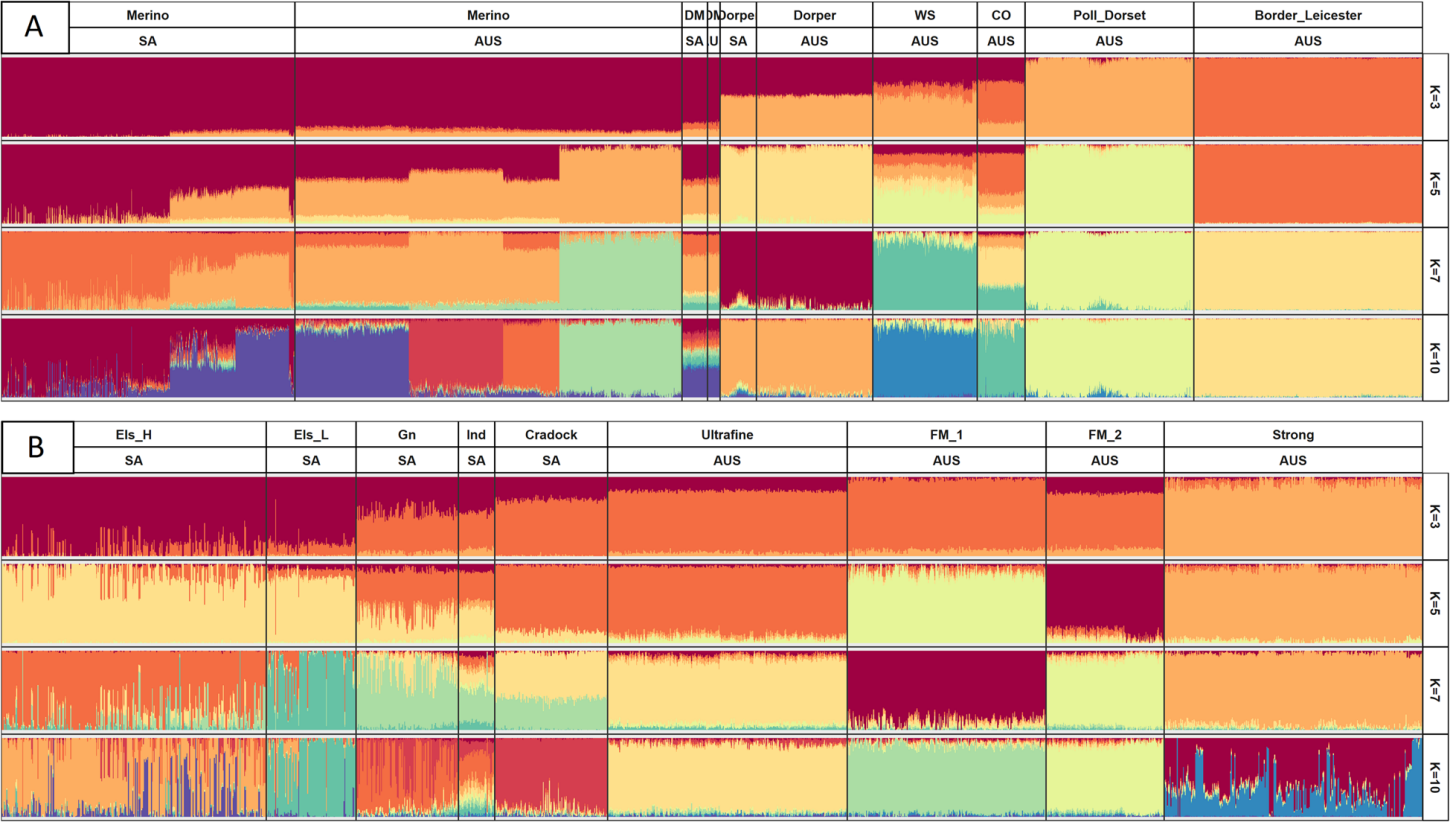
Stacked bar plot where each colour represents the proportion of a genetic group (Q) of an animal’s genome resulting from ADMIXTURE analysis for (**A)** breed groups and (**B)** Merino bloodlines. Breed labels of **A** are, in order: Merino, Dohne Merino, Dorper, White Suffolk, Coopworth, Poll Dorset and Border Leicester. Merino bloodline labels of**B** are, in order: Elsenburg High, Elsenburg Low, Grootfontein, Industry, Cradock, Ultrafine, Fine-Medium-1, Fine-Medium-2 and Strong

Model-based clustering by ADMIXTURE into genetic groups generally revealed genetic compositions unique to the respective population groups (Fig. 5). Assignment of genotypes at K = 3 had a definite separation of Merino and Border Leicester groups (Fig. 5a). The dual-purpose Dohne Merino groups clustered with the Merino groups, while the Dorper and White Suffolk clustered with mixed composition of both Merino and Poll Dorset co-ancestry. The Coopworth group was separated into almost equal proportions of all three groups. At K = 5, within-group substructures became distinct in both AUS and SA Merino groups and were increasingly defined when analyzed at higher values of K. The Dorper, Poll Dorset and Border Leicester breeds appeared distinct as individual groups, while the admixed history of the Dohne Merino, White Suffolk and Coopworth groups was reflected by assignment to multiple genetic groups. At K = 7, the Coopworth appeared admixed with links to White Suffolk, Merino, and Dorper, but predominantly remained clustered with the Border Leicester. At K = 10, most breed groups were genetically distinct except for the Dohne Merino, whose composition coincided with multiple genetic groups within the Merino cluster of breeds.

An additional set of runs of ADMIXTURE for Merino only genotypes are also reported (Fig. 5b). At K = 3, bloodlines appeared similar except for the Elsenburg and Strong lines which showed no apparent co-ancestry. The genetic composition of Elsenburg line displayed low levels of heterogeneity, which increased with subsequent values of K. At K = 5, the Elsenburg, Fine-Medium and Strong lines were predominantly divided into distinct groups. The group proportions of the Ultrafine line were very similar to the Cradock line, which as a group, shared notable genetic proportions with the Grootfontein and Industry lines. At K = 7, the shared genetic composition between the Ultrafine and Cradock lines persisted. However, this was not the same group that linked the three SA lines - Grootfontein, Industry and Cradock, suggesting that different pathways of gene flow connected these bloodlines. At K = 10, the Elsenburg, Grootfontein and Strong lines appeared considerably more heterogenous in contrast to the remaining groups. The Fine-Medium, Ultrafine and Cradock lines were grouped into distinct groups with small proportions of contradicting ancestry. For both the full (by breed) and subset (by bloodline) analyses, the ADMIXTURE cross-validation statistic for K = 3 to 20 is reported in Fig. 5b. For this range, increasing numbers of clusters (i.e. level of K) resulted in an increased accuracy (lower CV statistic), however, a minimum value of CV was not observed for the 3 to 20 K values investigated.

### Linkage disequilibrium and effective population size

From the non-syntenic LD of randomly sampled groups, a strong relationship with 1/N was observed, indicating that measures of LD is rapidly inflated as the sample size decreased below N = 64. This trend can be seen in Fig. S1, which also shows the correction factor suggested by Hill & Robertson [9]. Alternatively, a simple correction: ${r}^{2}- \frac{1}{N}$ could have been effective but would inevitably lead to negative *r*^2^ estimates at very small sample sizes and low levels of ${r}^{2}$. Consequently, LD and effective population size (N_e_) is only reported for groups consisting of samples sizes larger than 64, but an exception was made for the SA ‘Industry’ group (N = 41), and results for this group should be treated with precaution.

**Fig 6:**
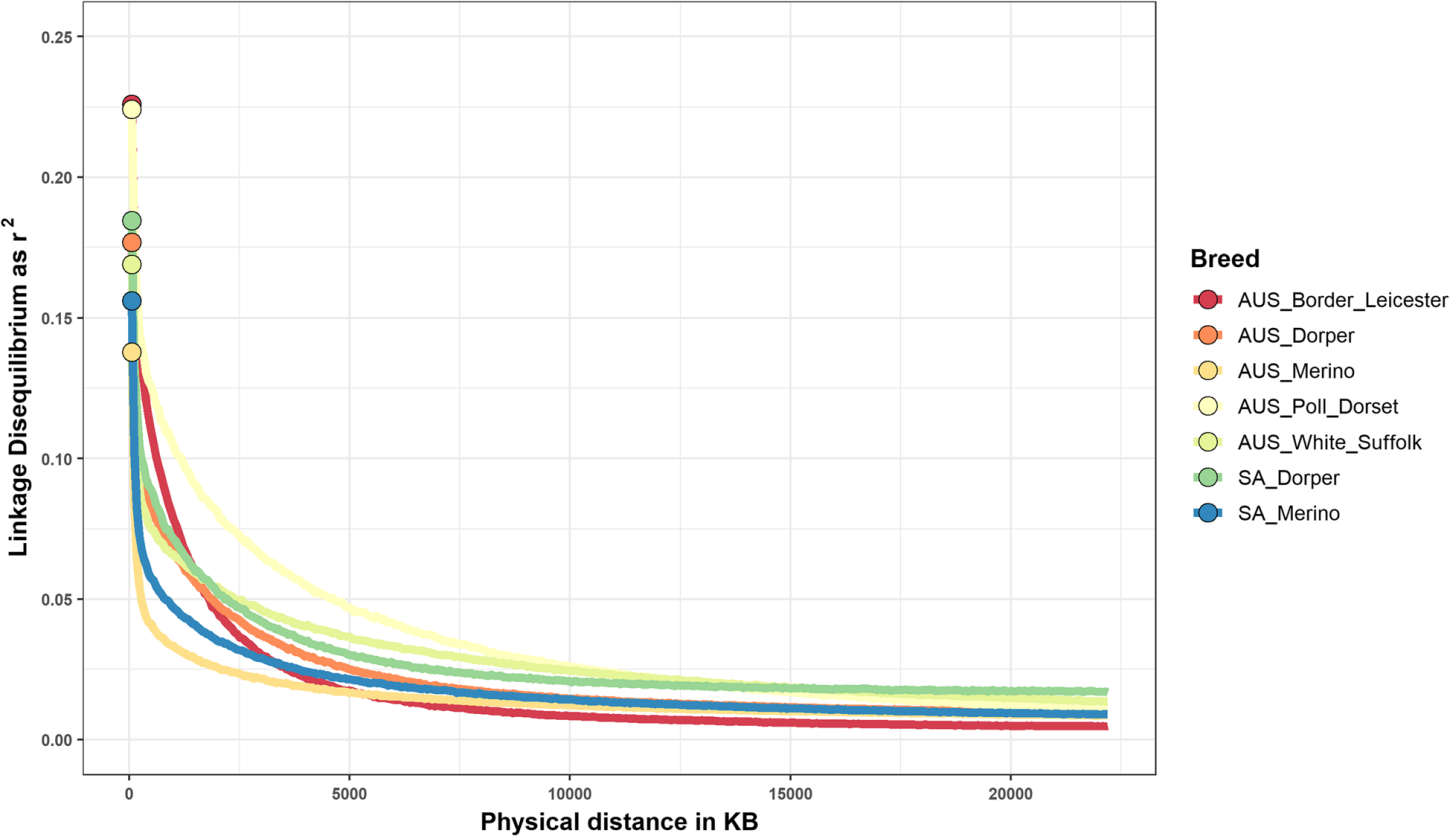
LD decay as levels of mean r^2^ over physical distance for selected breed groups. Dots represent the mean adjacent LD at ~ 55KB

The estimates of average LD between adjacent markers (separated by ~ 55 KB) showed substantial variation in the breed and bloodline groups. The lowest LD values were estimated for the AUS Merino (0.138) and second-lowest for the SA Merino (0.156). The highest LD values were estimated for the Poll Dorset (0.224) and Border Leicester (0.226) breeds. The persistence of LD over physical distance was different for each breed group (Fig. 6). The AUS Poll Dorset and Border Leicester had similarly high estimates of LD at the shortest interval of 55 KB, but a sharp distinction was observed in the patterns of LD decay between these two breeds. LD in the Poll Dorset group displayed a comparatively slow rate of decay and retained the ranking of highest LD to roughly 15,000 KB, at which point LD decay had stabilized for most other groups. The Border Leicester, in turn, was observed to have a rapid rate of decay between adjacent LD to a distance of 5000 KB, as its comparative ranking changed from the highest values of ${r}^{2}$ at 55 KB (adjacent) to close to being the lowest at 5000 KB, and persistently the lowest after 7500 KB. Across-country comparisons for the Merino and Dorper groups appeared to have a generally similar pattern of LD-decay, but with small differences at the absolute level. The AUS and SA Merino displayed the sharpest initial rate of decay trending noticeably lower than the remaining groups at initial distances.

**Fig 7:**
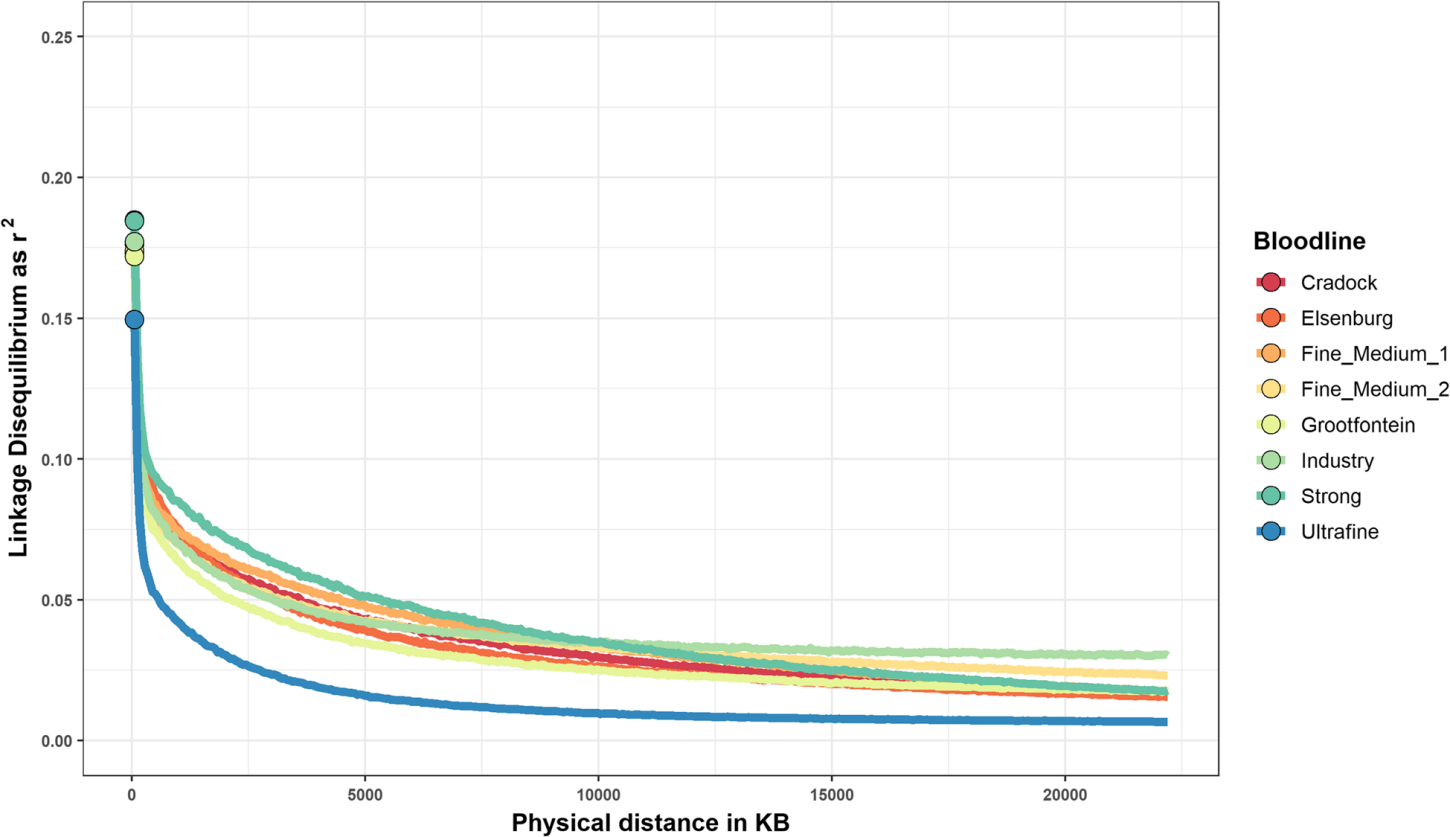
LD decay as levels of mean r^2^ over physical distance for selected Merino bloodline groups. Dots represent the mean adjacent LD at ~ 55KB

Patterns of LD decay were generally uniform between Merino bloodlines (Fig. 7), except for the rapid LD decay observed in the Ultrafine line, which was a persistent anomaly compared to the other bloodlines across all distances. In general, the split of Merino lines into sub-population by bloodlines had marked effects on LD trends. Excluding the Ultrafine line, the adjacent ${r}^{2}$ estimate for bloodlines were all above 0.17, which was noticeably higher than the values observed for both SA and AUS Merinos. Observing the pattern of decay (Fig. 7), the lowest bloodline estimate of ${r}^{2}$ was roughly 0.05 at a distance of 2500 KB, which was two-fold that observed for the AUS Merino at the same distance, and if excluding the Poll Dorset, higher than most breed groups at the same distance (Fig. 6).

**Fig 8:**
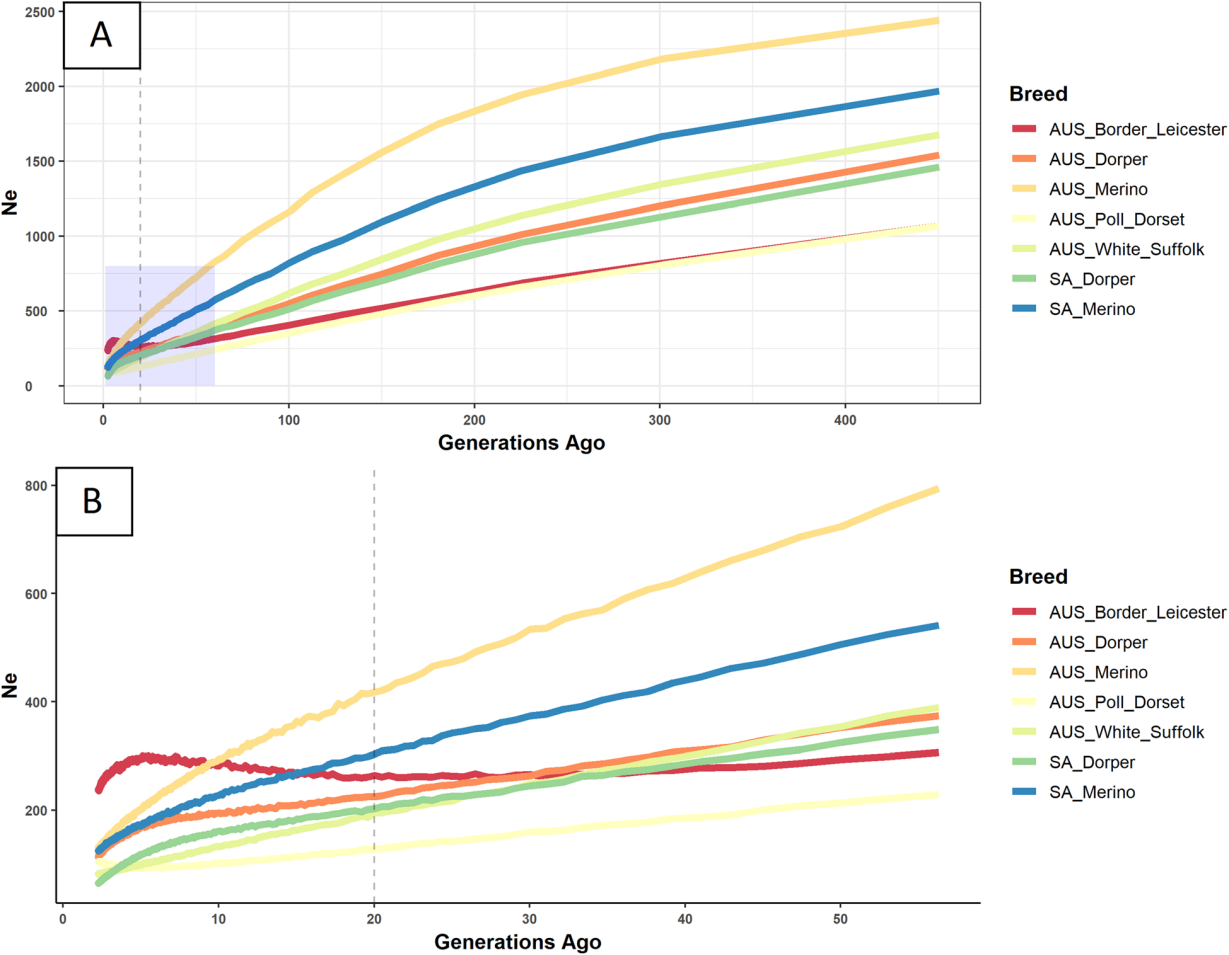
Historical effective population size (N_e_) for selected breed groups from (**A)** 2 to 400 generations ago and (**B)** 2 to 60 generations ago. In **A** and **B**, the vertical-line represents the timepoint used for ‘current’ N_e_. The transparent square on **A** represents the relative boundaries of **B**

The plotted estimates reflected N_e_ from 2 to 500 generations ago (Fig. 8). Uncharacteristic trends of N_e_ were observed for some breeds in the recent generations, and the ‘current’ effective population size was estimated at ~ 20 generations ago to represent diversity that was putatively considered a more stable time point (Table 4; Fig. 8). At this point, the AUS Merino was the most diverse population group with a N_e_ of 418, more than 3 times the N_e_ of 128 observed for the Poll Dorset group. The levels of diversity were more uniform across bloodlines, except for the Ultrafine line which had a large N_e_ estimate of 369 (Table 4). According to the estimates at this timepoint (in generations), the Ultrafine group has an effective size larger than multiple breed groups. In the historical trends of N_e_ for breed groups (Fig. 8) and bloodlines (Fig. S4), groups were generally consistent in their ranking across generations. The AUS Merino had a large historical population, but also the steepest rate of decline, especially from 200 generations ago. The SA Merino and Dorper groups were intermediate while the Poll Dorset and Border Leicester had relatively low historical population sizes with an N_e_ of just over 1000 at 400 generations ago. A notable exception in the N_e_ trends was observed over recent generations. From 20 generations ago, the Border Leicester group unexpectedly increased in N_e_. For the Merino bloodlines, the Ultrafine line was a recurring exception and maintained a persistently high N_e_ across the estimated time scale. The bloodlines displayed levels of diversity comparable to that observed for breed groups.

**Table 4:** Linkage Disequilibrium (LD) (adjacent (~ 55 KB) and non-syntenic) and effective population size (N_e_; determined 20 generations ago) for the selected breed and bloodline groups

Population		*N*	LD_*Adjacent*_	LD_*Non−Syntenic*_	N*e*_*20 Gen*_
*Breed*	*Country*				
** Merino**	**AUS**	918	0.138	0.006	418
** Dorper**		276	0.177	0.008	225
** Border Leicester**		542	0.226	0.003	265
** Poll Dorset**		400	0.224	0.005	128
** White Suffolk**		247	0.169	0.006	196
** Merino**	**SA**	697	0.156	0.006	304
** Dorper**		86	0.184	0.016	203
*Bloodline*	*Country*				
** Ultrafine**	**AUS**	270	0.150	0.005	369
** Fine-Medium-1**		224	0.173	0.009	154
** Fine-Medium-2**		133	0.174	0.016	178
** Strong**		291	0.185	0.009	137
** Elsenburg**	**SA**	400	0.185	0.010	173
** Grootfontein**		115	0.172	0.013	201
** Industry**		41	0.177	0.028	177
** Cradock**		127	0.176	0.011	165

## Discussion

### Heterozygosity and inbreeding

The current results for ${H}_{obs}$ were consistent with previous findings for the AUS Merino [13, 15, 16], SA Merino [21], Coopworth [14], Poll Dorset and White Suffolk [13] (Table 1). Current values were marginally higher than those previously reported for the African Dorper [13] and Border Leicester [13, 15], but the general ranking of the Merino, Poll Dorset and Border Leicester as breeds of respectively high, intermediate and low gene diversity agreed with previous results from marker data [13, 15, 22]. A similar range of 0.33 to 0.38 has been reported for mean heterozygosity of New Zealand pure and composite populations [14, 23] that originated from programs that included high levels of crossbreeding. Diversity of ‘pure’ breeds currently investigated could be expected less diverse compared to highly crossbred populations, suggesting either paricularly high diversity for current breed groups or that ${H}_{exp}$ and ${H}_{obs}$ has limited sensitivity as a measure of genetic diversity.

In deriving estimates of inbreeding by marker-based methods, it is important to account for the original definitions of inbreeding described as the correlations between homologous genes within a haploid individual [24], or the probability of identity by descent [25]. The former definition accommodates a negative F-value [26]. The latter does not as probabilities, by definition, are bounded between 0 and 1. Also, estimates of inbreeding derived from the diagonal of the GRM are sensitive to the extent that breed effects influence allele frequencies. The mean (1.13) of the diagonal of $\boldsymbol{G}$ suggested the multi-breed composition of this GRM slightly inflated estimates of F_VR_ across all groups. This is supported by the fact that F_VR_ estimates of Merino bloodlines, derived from ${\boldsymbol{G}}_{M}$ (mean diagonal of 1.05), are slightly lower than the mean F_VR_ for Merinos according to $\boldsymbol{G}.$ These estimates of F_VR_ could thus be considered appropriate for comparing mean levels across groups within the same GRM, but represent slightly inflated measures of individual inbreeding.

The high mean F_VR_ suggested relative uniformity within populations such as the Dorper, SAMM, Poll Dorset and Border Leicester groups. The high levels of inbreeding for the AUS Dorper and AUS SAMM could be expected as these breeds were established in Australia from limited importation of genetic material from South Africa followed by grading-up programs using back-crossing. Also, F_VR_ was similar or higher in the SA populations, implying that inbreeding in the AUS populations was also a characteristic of their ancestral lines, rather than only a consequence of across-country isolation. High levels of inbreeding precipitate a decline in quantitative genetic variance [27], and detrimental effects associated with excess homozygosity have been reported in sheep [28–30]. The overall level of inbreeding in populations observed here was generally low, but the comparatively high values for breeds such as the Border Leicester and Poll Dorset are notable for consideration in their breeding program design.

### Pairwise F_ST_ statistics

Given analogous definitions for F_ST_ statistics [31, 32], F_ST_ estimates can be thought of as (1) the correlation between randomly sampled alleles within subpopulations relative to the total population or (2) the proportion of genetic variance that can be attributed to variance in allele frequencies between subpopulations [33]. With ‘across breed’ estimates ranging from 0.05 to 0.26 (Table 2), it was clear that the implications of this segregation depend greatly on the historic admixture between any two distinct breeds. Current F_ST_ values were higher than previous estimates of 0.062 and 0.053 between the Merino and, respectively, the Poll Dorset and Border Leicester groups [15], but methods were not similar. The low levels of divergence across countries for the SAMM, Dorper and Dohne Merino breed groups reflects the formation of these breeds in AUS by the importation of genetic material directly from SA. The low estimate for SA x AUS Merino groups suggested that the two Merino populations have not diverged greatly, or that the few recent genetic links were influential in reducing the genetic distance of samples included in this study. Interestingly, some F_ST_ values between bloodlines were comparable or larger than certain across breed estimates (Table 3). This further highlights the importance of lines that have already been noted for AUS Merinos [19], and also suggests that partial restriction of gene flow could have important implications, regardless of being ‘within breed’.

The low level of divergence between the Ultrafine and Cradock groups is somewhat expected since these bloodlines were known to have recent across-country links. However, the F_ST_ estimate in this across-country comparison was as low as pair-wise comparisons between SA lines, which is promising for the prospect of a common genomic evaluation for these populations.

### Linkage disequilibrium and effective population size

Overall, the reported LD at ~ 55 KB agreed with previous findings that characterised LD in sheep to be generally low [22] compared to other domesticated species such as pigs [34] and dairy cattle [35]. Current results for Merinos agreed with earlier reports that characterized this breed by rapid LD decay [15, 22, 36]. However, Kijas et al. [22] showed substantial differences in LD measurements when determined at shorter distances such as ~ 10 KB, and higher density platforms could thus provide a more accurate indication of LD decay than currently reported.

LD observed for bloodlines reflected the importance of structuring by subpopulations. When compared to Merino breed groups, which is the pooled bloodlines, the pattern of decay showed relative consistency, but the absolute level of LD was substantially higher when structured by subpopulation (Fig. 6 vs. Fig. 8). Thus, while overall levels remained low, the persistence of LD was noticeably sensitive to the connectedness of set populations. Extrapolating this pattern, it could be speculated that the Ultrafine line consists of influential population substructures not accounted for by the current population assignment, which is supported by the low level of relatedness, but the slightly higher F_VR_ within the Ultrafine line. However, no obvious substructures were observed in either PCA or ADMIXTURE results, as discussed below, and it is thus difficult to explain the uncharacteristic pattern of decay of this bloodline.

Calculating historical N_e_ by the rate of LD decay [37] has numerous examples in sheep [13, 14, 23]. Domestic populations, under strong selection programmes with the widespread use of preferential sires, deviates heavily from the assumption of random mating of Wright-Fisher populations [38] and the loss of diversity seen in Fig. 8 is expected. However, compared to other domestic species, sheep have been characterized by relatively high levels of diversity [13], attributed to large founding populations combined with less intense selection compared to other domestic species, such as cattle [39]. Using the same methodology, Kijas et al. [13] reported roughly similar estimates for African Dorper (264) and Border Leicester (242), but considerably higher estimates for the Australian Merino (833), Poll Merino (918) and Industry Merino (853) and Australian Poll Dorset (318) which indicates some inconsistency in N_e_ estimates of the same breeds.

 The unexpectedly increasing trend of N_e_ over recent generations seen in Fig. 8(b) made it difficult to evaluate the breed’s comparative ranking across more recent generations. Other studies have also reported increasing N_e_ estimates in recent generations. Using a similar methodology, Brito et al. [23] reported an increasing N_e_ for the Primera and Lamb Supreme breeds over the last 5 generations. An increase in N_e_ was also observed for the Romney breed around 20 generations ago only to decline at around 5 generations ago [14]. The latter authors ascribe the increase in N_e_ to an increase in animal numbers following successful management and the recent implementation of technologies like artificial insemination [14]. However, non-random mating reduces N_e_ below census size, N [38]. Thus, given the high intensity of artificial selection and genetic drift, a population-wide increase in genetic diversity is unexpected in the absence of crossbreeding. Also, according to Hill & Robertson [9], LD and fixation have a linear relationship. Given that the Border Leicester group had the highest F_VR_ and within-group relatedness, it was surprising not to observe a pattern of LD, and consequently N_e_, more comparable to less diverse breeds such as the Poll Dorset group.

### Genomic relatedness, PCA and ADMIXTURE

Estimates of relatedness according to $\boldsymbol{G}$ agreed with F_ST_ estimates in describing a close association between breeds in common between countries. The across-country relationships for the SAMM and Dorper breeds were only marginally weaker than the internal relatedness of individual populations (within country). The lower level of relatedness between the AUS and SA Dohne Merino populations could be due to a more diverse SA population, as the SA Dohne Merino maintained a lower F_VR_ and internal relatedness compared to the SA SAMM and SA Dorper groups.

For the Merino breed, the across-country relationship is known to be connected by both deep ancestral and relatively recent relationships, but the positive relationship was expected to be low for multiple reasons. Both groups were internally diverse, and it can be reasoned that a population cannot be more related, on average, to another population than its internal level of relatedness (than it is to itself). Also, the ancestral relationship between SA and AUS Merinos is defined by a distant linkage in the original development of the AUS Merino [4]. Lastly, the known recent links are limited to those between the Cradock and Ultrafine lines. Considering the defining influence of structure between bloodlines, this across-country relationship is likely to be isolated within these two lines, a tendency also observed in the ADMIXTURE, PCA and F_ST_ results reported here.

This study also reported across breed relationships that were comparatively high in magnitude. These cannot be attributed to recent links and must thus be prompted only by a deeper co-ancestry. For example, the SAMM and Dohne Merino share origins to the German Mutton Merino [5], and the origin of the AUS Coopworth is linked to the Border Leicester (www.coopworth.org.au).

From ${\boldsymbol{G}}_{\boldsymbol{M}}$, the positive relatedness between SA bloodlines suggested that separation on a flock level was less restricting compared to that of lines or strains, which was expected. The generally weak relationships with the Elsenburg line reflect the initial management on an isolated basis. The positive relationship between the Grootfontein and Industry lines is a good indication of the resource flock’s objective to represent commercial SA Merinos. Given the relatively few genetic links between the Ultrafine and Cradock lines, it was surprising to observe this across-country relationship as the only positive relationship in a comparison involving AUS bloodlines, including relationships to other AUS Merino bloodlines within country.

Results from PC- and ADMIXTURE analysis generally indicated very similar clusters of breed and bloodline populations. The discrete breed structures of PC1 and PC2 appeared to capture deep ancestral relationships and very little within-group variation. From ADMIXTURE analysis, the distinct genetic structures of Merino, Border Leicester and Poll Dorset agreed with those three breeds occupying the most distant branches of PC1 and PC2.

The ADMIXTURE and PCA results also agreed well with previous parameters that indicated only small effects of across-country separation. However, a study that imputed SA breeds from AUS reference panels showed a markedly higher accuracy of imputation for Dorpers compared to Dohne Merinos [40]. Although imputation accuracy is not directly related to ADMIXTURE and PCA results, it is thought that the homogenous nature of the Dorpers suggested by Fig. 5 is likely to have facilitated the more accurate imputation for this breed.

Generally, breed structures did not dissipate in succeeding principal components as groups remained clustered beyond PC1 and PC2 (Fig. S2), and the genotypes of most breeds were defined by a similar genetic composition in ADMIXTURE analysis (Fig. 5a). However, Merino groups were an exception and often segregated into multiple clusters across PC3 to PC14 and substructures of Merinos were also clear from ADMIXTURE analysis that commenced on an ‘across breed’ level. Following the advent of artificial insemination (AI), across bloodline links are considered to have become more common in AUS [18], but these results strongly suggested that the subpopulation, i.e., bloodline of origin, is an important determinant of the genetic composition of Merinos. This has been demonstrated by high levels of quatitative genetic variance across similar groupings (Ultrafine, Fine/Fine-medium, Medium/Strong) of AUS Merinos for key production traits [18]. Also, markedly different accuracies for genomic breeding values were reported for similar groupings (superfine-, fine- and strong-wool types) following genomic prediction of production traits from the same reference set [41].

The ADMIXTURE analysis of bloodlines revealed further complexities of these population structures (Fig. 5b). The identification of the Ultrafine and Strong lines within opposing clusters have been previously reported [17]. Similar to other metrics presented in this study, close association existed between the Cradock line and the Ultrafine and Fine-Medium-1 lines. However, ADMIXTURE results at K = 7 suggested that the remaining SA lines might not directly benefit from the relationship between the Cradock and Ultrafine lines. These results should also be seen in combination with the discussion below with reference to POV and the CV error.

The highly defined population structure of the Ultrafine line is notable considering the high estimates of diversity according to N_e_ and relatively low internal relatedness. However, this is possibly due to the orthogonal nature of principal components, which separated the ancestral relationships for which the Ultrafine line appears uniform, from within-line variation between sampled individuals. This could also partly explain the homogenous composition observed in ADMIXTURE analysis. If the Ultrafine line did consist of many small substructures as previously speculated in this paper, the level of K was likely too low to capture such structures for a population with a strongly defined ancestry.

Despite the good agreement and accuracy of PCA and ADMIXTURE analysis in identifying the known groups of origin, the low POV explained by the initial principal components in Fig. 5a supports previous results that specifically noted the high dimensionality as a characteristic of genetic architecture of sheep [13, 42]. Also, the lack of an inflection point in CV errors from 3 to 20 (Fig. S5b) implied that the model had difficulty estimating an ideal value of K within this range. It is possible that higher levels of K would perpetually identify lower-level structures, such as families or sire groups, as unique genetic groups. The high diversity of the animals in this study and in sheep in general could exacerbate this problem, causing difficulty in estimating a ‘best’ estimate for K. Thus, further analysis across higher increments of K were not explored, also because a similar pattern has been observed for similar sheep datasets with fewer breeds until K = 40 (P. Gurman, unpublished data).

### Implications for genomic selection

The combining of populations into the same pool - such as the current scenario of merging bloodlines into respective SA and AUS Merino groups - hopes to benefit prediction by increasing sample size. In the presence of heterogeneity this will be accompanied by an associated increase in N_e_, and thus a decrease in LD. This trade-off could be an important determinant in breeding program design, and the more diverse and diverged the populations, the more challenging this trade-off is likely to be. Thus, an alternative consideration is also valid that a population of high diversity could benefit from being subset into smaller groups of better-connected animals. Regarding genomic selection, Van der Werf et al. [11] showed that a small number of highly related individuals could be more informative than large numbers of distant individuals. While this previous study binned relatedness by categories (e.g. groups of half-sibs), it should also be valid across a continuous scale of heterogeneity such as currently seen in comparing combinations of populations. In a narrow spectrum approach, knowledge of important population structures would be essential to identify pockets within the population that could deliver optimal results. For a subset of Merino populations such as the Cradock and Ultrafine lines, the mean relatedness was as high as the mean internal relatedness for AUS Merinos, which are all currently evaluated in a single analysis [2]. Further measures of PCA, ADMIXTURE and F_ST_ indicated that these two groups are likely to be the best starting point for an across-country platform. However, these ‘bloodlines’ were the only examples of some, albeit low, linkage by pedigree. A minimum level of genetic exchange is thus likely to remain an important factor unless more distant population structures are better accounted for in future evaluations.

Accounting for population structures derived from the extended pedigree have delivered increased accuracies for predicted genomic breeding values in the AUS Merino [17]. Including eigenvalues from PCA analysis [42], or group proportions from the ADMIXTURE Q-matrix [17], has decreased accuracy, but the higher accuracy of non-adjusted values are likely biased by picking up on breed effects rather than individual variation. Initially, adjusting for population gene frequencies showed little benefit [12], but improved results have recently been reported by Gurman et al. [43] for a multi-breed GRM. Given that both PCA and ADMIXTURE results proved informative and accurate in characterizing populations by known group of origin, further research is needed to make efficient use of this information. However, other problems could persist in the likely case where across country prediction would utilize both pedigree and genomic information in the ‘single-step’ approach [44] which is now common for Australian sheep [2]. The assumption of unrelatedness of founder parents in the pedigree could be particularly problematic in the case of across country separation where disjoined pedigrees could in fact be well connected by unknown links. In this regard, the use of so called ‘metafounders’ [45] could be a promising approach to better align the pedigree and GRM for both disconnected and highly related base populations, but depends on all genetic groups being well represented in the genotypic dataset.

## Conclusions

These results provide valuable information on the population structures of important sheep breeds in South Africa and Australia. According to multiple parameters, the isolation across some bloodlines was as influential as that observed across breeds and showed that important division into subpopulations can be extended beyond breed structures. The connectedness of SA resource flocks suggested they could be valuable for contributing to a pooled SA reference population in combination with industry animals. In the prospect of across-country amalgamation, isolation by country rarely proved restricting. While the need for genetic links remains important, the generated knowledge delivers potential to maximize the benefit of such relationships by a narrow spectrum approach to combining populations. However, the wide range of characteristics observed across and within breeds, bloodlines and flocks suggests that the optimal approach would be unique to any given set of populations and breeding objectives. Investigating across-country genomic prediction and imputation of these populations is recommended.

## Methods

### Sample populations

This study examined 3509 genotypes, which are summarised by breed and country in Table 1. The selected AUS genotypes in this study were a group of samples used as training animals for supervised ADMIXTURE [46] analysis in a previous study [20]. The genotypes selected by Gurman et al. [20] were intended to capture the diversity in the Australia sheep population. The SA samples were obtained from resource flocks maintained by institutions across the Western Cape and Eastern Cape provinces of South Africa [5] as well as industry animals from commercial lines whose records are managed by the National Small Stock Improvement Scheme. The latter group of commercial South African genotypes are herewith referred to as ‘Industry’. For this study, the non-Merino SA breed groups originated from either industry or resource flocks: Dorper (Nortier = 20, Industry = 66), Dohne Merino (Industry = 39, Langgewens = 12, Elsenburg = 9), SAMM (Industry = 36, Nortier = 21), and Dormer (Industry = 32, Elsenburg = 10) were pooled under their breed name. Additionally, SA and AUS samples within the Merino breed were subset according to bloodline and/or the flock of origin and separately analysed as a set of Merino sub-populations (Table 1). The SA Merinos were separated on a resource flock level and the AUS Merinos by strain (or line), but the subpopulations from both SA and AUS are henceforth collectively referred to as ‘bloodlines’ for simplicity.

The AUS Merinos are historically grouped into the bloodline of origin, partly by performance in fibre diameter, but also by region, with the ‘fine’-type strain popular in high rainfall zones, the ‘medium’ in cropping zones and the ‘strong’ more common in drier pastoral areas [18]. The Elsenburg Merino flock is a resource flock that is divergently selected for reproductive performance by the number of lambs weaned per ewe mated. The flock is subset into two lines, the H-Line (positive selection) and the L-Line (negative selection); [47] and was maintained as an isolated group from 1986 until the first inclusion of external sires in 2008 [48]. The Grootfontein Merino stud is a research flock managed according to commercial objectives and is the most traditional representation of South African commercial lines by a resource flock. It was subjectively selected for ‘overall excellence’ from 1968 to 1985 [49] whereafter objectives changed to increasing live weight and decreasing fibre diameter while maintaining fleece weight [50]. The Cradock fine wool Merino stud was established in the 1980s when Merino ewes of different flocks were screened into a central flock and mated to four AUS fine wool sires for two years. This was followed by the using two more AUS sires in 1996 and another two ‘Ultrafine’ rams in 2002–2003 [51]. Selection objectives were first defined by increased live and fleece weight from 1988 to 1996 followed by an increased emphasis on reducing fibre diameter since 1996.

### Experimental design

Analyses were firstly conducted for (n = 13) subpopulations defined by breed and country of origin (for common breeds), with combinations examined. Second, analyses were repeated only for Merinos with populations defined by the (n = 9) bloodlines as described above. However, the ‘Langgewens’ group was often excluded from analyses due to the small number of genotypes, and the fact that this small subgroup was not pertinent to the outcome of the study as representing SA Merinos.

### Genotyping and quality control

Genotyping was performed using the OvineSNP50 Chip (Illumina Inc., CA, USA). Quality control measures were applied to SNPs (GenCall score > 0.25, GenTrain score > 0.5, MAF > 0.01, call rate > 0.95) and samples (call rate > 0.95). Randomly missing SNPs were imputed with ‘FIMPUTE’ (Version 2.2) [52] and ‘Beagle’ (Version 4.0) [53] software for SA and AUS samples, respectively. Following quality control and imputation, the combined dataset consisted of 47,789 markers on 3,509 animals, with an average marker spacing of roughly 55 kilo-basepairs (KB). Genotypes were called according to the AB system and numerically coded according to B-Allele content (i.e., 0, 1 or 2) unless other formats were required.

### Genomic analysis

Mean allele frequencies within populations were used to derive expected and observed levels of heterozygosity as summary statistics of overall gene diversity. Proportional differences in frequencies between subpopulations were represented by F_ST_ statistics as measures of divergence. Relationships based on the genomic relationship matrix (GRM) [54] and decomposition of the GRM by principal component analysis (PCA) were used to investigate relatedness between individuals across and within population groups. Additionally, ‘ADMIXTURE’ [46] was used to estimate the extent to which individuals identify to the same ancestry based on shared chromosome segments. Linkage disequilibrium (LD) was used to express the correlation between markers in a discrete population and was also used to estimate recent and historical effective population size (N_e_). Relationships between more closely linked markers tend to reflect deeper histories since it would require an increasing number of generations for recombination to segregate markers more closely linked [55]. Furthermore, Hayes et al. [37] showed that the relationship between markers at a given distance can be used to estimate N_e_ at a given number of generations ago, which can be used to deduce the population history by trends in the effective population size. Unless stated otherwise, plots were prepared in the ‘ggplot2’ ‘R’ package [56].

### Heterozygosity and F-statistics

The expected (${H}_{exp}$) and observed heterozygosity (${H}_{obs}$) were calculated for each marker. The mean of all markers and the respective standard deviations across markers were reported for all population groups. ${H}_{exp}$ was calculated as expected under Hardy-Weinberg equilibrium: ${H}_{exp}=2\left({p}_{i}\right)\left(1-{p}_{i}\right)$; where *p*_*i*_ is the frequency of the *i*_*th*_ allele in a defined population. Genetic distances between populations were determined by pairwise F_ST_ statistics which was calculated for each marker, and averaged over all markers by deriving $a$, $b$, and $c$ according to Weir & Cockerham [31] and summing variance components across $i$ markers separately:

$${F}_{ST}={\theta }_{W}= \frac{\sum _{i}{a}_{i}}{\sum _{i}({a}_{i}+ {b}_{i}+ {c}_{i})}$$

All of these analyses were performed using custom scripts in ‘R’ (Version 3.6.1) [57].

### Genomic relationships, inbreeding and principal component analysis

Genetic relationships were examined by calculating the genomic relationship matrix (GRM) in R according to VanRaden [54]:

$$ \boldsymbol{G}=\frac{\left(\boldsymbol{M}-2\boldsymbol{P}\right)\left(\boldsymbol{M}-2\boldsymbol{P}\right)`}{2{\sum}_j\left({\boldsymbol{p}}_j\right)\left(1-{\boldsymbol{p}}_j\right)} $$

where $\boldsymbol{M}$ is the genotypic matrix of marker counts with dimensions *m* x *n* (number of genotyped animals, *m*, by number of markers, *n*) and $P$ is the corresponding matrix (*m* x *n*) where the *j*th of $\boldsymbol{P}$ is a vector of *m* replicates of the allele frequency ${p}_{j}$ within all the animals in the analysis. The diagonal of the GRM was used to calculate genomic inbreeding coefficients (F_VR_) as ${\boldsymbol{G}}_{ii}-1$, with the population inbreeding reported as the mean of the inbreeding coefficients. The overall relatedness between or within populations was calculated as the mean of the off-diagonal of the GRM for the two groups of animals. For breed group comparisons, a GRM was calculated with all 3509 animals as $\boldsymbol{G}$, while Merino bloodline comparisons were derived from a GRM that only included the 1535 animals of Merino origin as ${\boldsymbol{G}}_{M}$. These values were visualized using the ‘corrplot’ R package [58] with the diagonal of the plot replaced by mean relatedness to all other animals in the GRM, but excluding internal relatedness to animals of the same group. A visual analysis of genetic relationships between and within populations was also performed by visualization the results of a Principal Component Analysis (PCA) of the GRM calculated using the ‘irlba’ ‘R’ package [59]. The first 40 principal components were calculated for both GRMs. In both cases, the first fourteen components (PC1 to PC14) were visualized to investigate between and within-group clustering. The cumulative proportion of variance (POV) explained by the first 40 principal components was also plotted.

### ADMIXTURE analysis

Genetic group proportions and ancestry for each of the samples were estimated by unsupervised runs of ADMIXTURE with a varying number of groups, K. ADMIXTURE produces a cross-validation statistic, ‘CV error’, as an indication of accuracy relative to the choice of the number of groups, K. The lowest CV error is considered the ‘best’ choice of K for determining proportions of shared chromosome segments from co-ancestry. ADMIXTURE analysis was repeated for K = 3 to K = 20 and results of K = 3, 5, 7 and 10 were chosen for visualization by stacked bar plots. This entailed plotting the proportions of each genetic group (Q) within an individual animal by a stacked bar graph. The CV error statistic for K between 3 and 20 was also plotted.

### Linkage disequilibrium and effective population size

Linkage disequilibrium (LD) between any two markers was calculated by:

$${r}^{2}= \frac{{\left({p}_{AB}- {p}_{A}{p}_{B}\right)}^{2}}{{p}_{A}{(1-p}_{A}\left){p}_{B}{(1-p}_{B}\right)}$$

where subscripts ‘A’ and ‘B’ is used to distinguish between the two pair-wise markers of a single *r*^*2*^ estimate (i.e. ${p}_{A}$ is the B-allele frequency of the first marker, ‘A’, and ${p}_{B}$ is the B-allele frequency of the second marker, ‘B’) and ${p}_{AB}$ is the frequency of the two B-alleles of the two pair-wise SNPs inherited together in a population. Pairwise LD was calculated at a depth of 400 markers using the ‘snpStats’ R package [60] (for each marker LD was calculated for the 400 markers that succeed it in genome order).

For the syntenic LD, the original genotypes involving the initial 47,789 markers were subset to only contain those needed to compute syntenic pairwise comparisons, leaving 400 pairwise LD estimates each for 37,789 markers. Subsequently, the means for pairwise LD estimates were used to determine mean LD for adjacent markers and subsequent LD decay using LD from SNP pairs of greater depth (gaps of 1 to 400 SNPs). A physical distance was assumed for LD decay by multiplying the depth of pairwise SNPs with the mean distance between adjacent SNPs (~ 55 KB). No attempt was made to estimate LD at intervals shorter than ~ 55 KB spacing, as this would rely on much fewer SNPs that are only sporadically associated by shorter distances. The LD procedure was repeated with a more stringent filter on MAF (> 0.1), but effects were negligible (results not shown). The pattern of LD decay over distance was plotted for breed and bloodline groups. For non-syntenic LD, results were subset to include only pairwise comparisons from different chromosomes and a single mean was calculated as there was no need to observe non-syntenic decay over distance.

To investigate the effect of sample size on LD estimates, the procedure for non-syntenic LD was repeated for a range of sample sizes (N = 2^n^ where n = 1 to 11.75) where a given population at given sample size N is sampled from the full genotype matrix at random.

LD estimates were used to calculate historical effective population size (Ne_t_) as:

$${Ne}_{t}= \frac{\left(1- {r}^{2}\right)}{4 c {r}^{2}}$$

where $N{e}_{t}$ is the effective population size at $t$ generations ago and ${r}^{2}$ is the calculated LD at each value of *c*, the recombination rate in Morgans. The values of $N{e}_{t}$ were calculated on the assumption of 1 Morgan = 100 million base pairs (MB) and $t =\frac{1}{2c}$ [37].

## Supplementary Information

**Additional file 1: Fig. S1. **Non-syntenic LD as r^2^ calculated from sample sizes of 2^n^ (where n ranged from 1 to 11.75) with the reciprocal of sample size (N) and the correction factor suggested by Hill and Robertson, (1968).

**Additional file 2: Fig. S2. **The third (PC3) to fourteenth (PC14) principal components of genotypes identified by breed group.

**Additional file 3: Fig. S3.** The third (PC3) to fourteenth (PC14) principal components of genotypes identified by bloodline.

**Additional file 4: Fig. S4.** Historical effective population sizes (Ne) for Merino bloodline groups from 2 to 400 generations ago. The vertical-line represents the timepoint used for ‘current’ N_e_

**Additional file 5: Fig. S5.** (**A**) Cumulative proportions of variance (POV) explained by the first 40 principal components of 'by breed' and 'by Merino bloodline' analysis. (**B**) The "CV-error" statistic resulting from sequential ADMIXTURE runs or K = 3 to K = 20 of 'by breed' and 'by bloodline' analysis.

**Additional file 6: Table S1** Mean relatedness across breed groups according to the genomic relationship matrix (***G***). Estimates in bold are the internal relatedness for a breed group calculated without the diagonal of (***G***).

**Additional file 7: Table S2** Mean relatedness across Merino bloodline groups according to the subset genomic relationship matrix (***G***_***M***_). Estimates in bold are the internal relatedness for a bloodline group calculated without the diagonal of (***G***_***M***_).

## Data Availability

The data that support the findings in this study are available from the various stakeholders including the South African Department of Agriculture, Land Reform and Rural Development, Western Cape Department of Agriculture, Western Cape Agricultural Research Trust, Meat and Livestock Australia and the CRC for Sheep Industry Innovation. Restrictions apply to the availability of these data, which were used under licence for this study, and is not publicly available. Data are available from the authors with the permission of the aforementioned stakeholders.
