# Supplementary figures and images for: The genomic structure of isolation across breed, country and strain for important South African and Australian sheep populations
Source: BMC Genomics. 2022 Jan 4;23:23. doi: 10.1186/s12864-021-08020-3 (PMC8725491; doi:10.1186/s12864-021-08020-3)

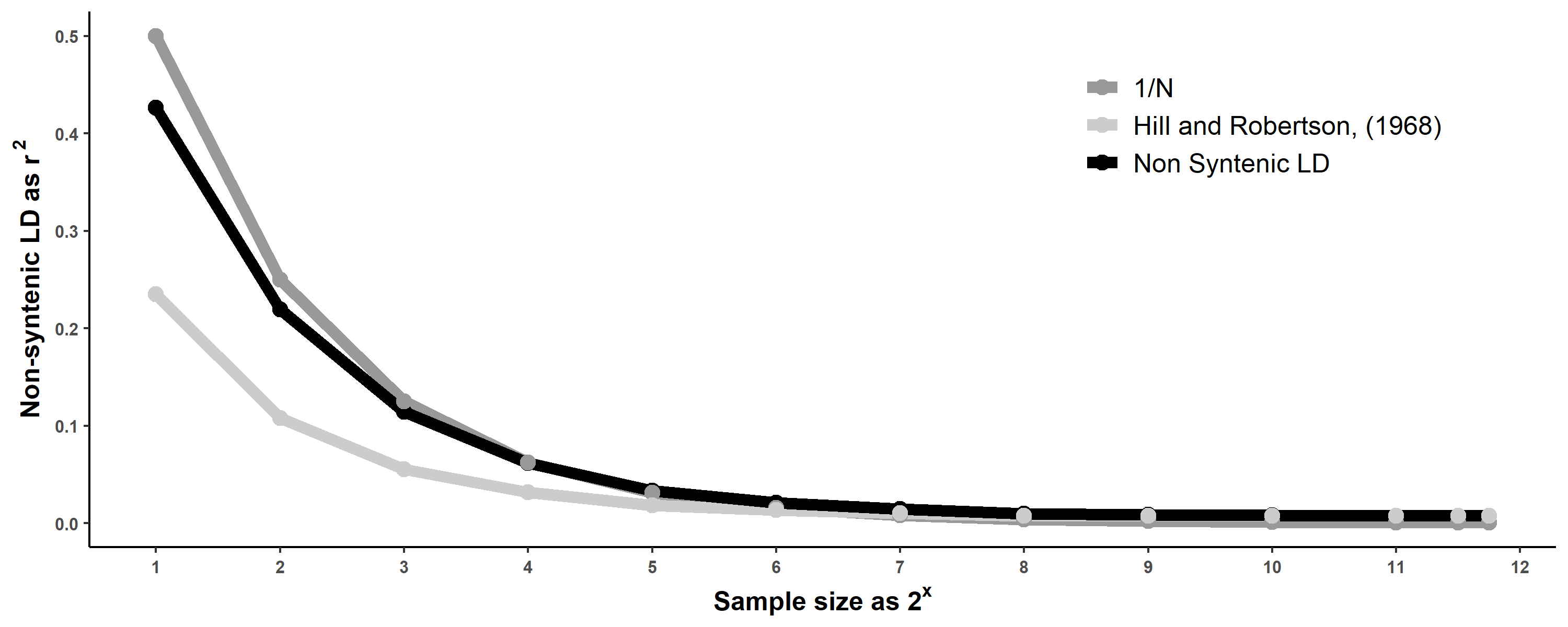

Supplement: Supplementary file 1 — Additional file 1: Fig. S1. Non-syntenic LD as r2 calculated from sample sizes of 2n (where n ranged from 1 to 11.75) with the reciprocal of sample size (N) and the correction factor suggested by Hill and Robertson, (1968). [file 12864_2021_8020_MOESM1_ESM.png]

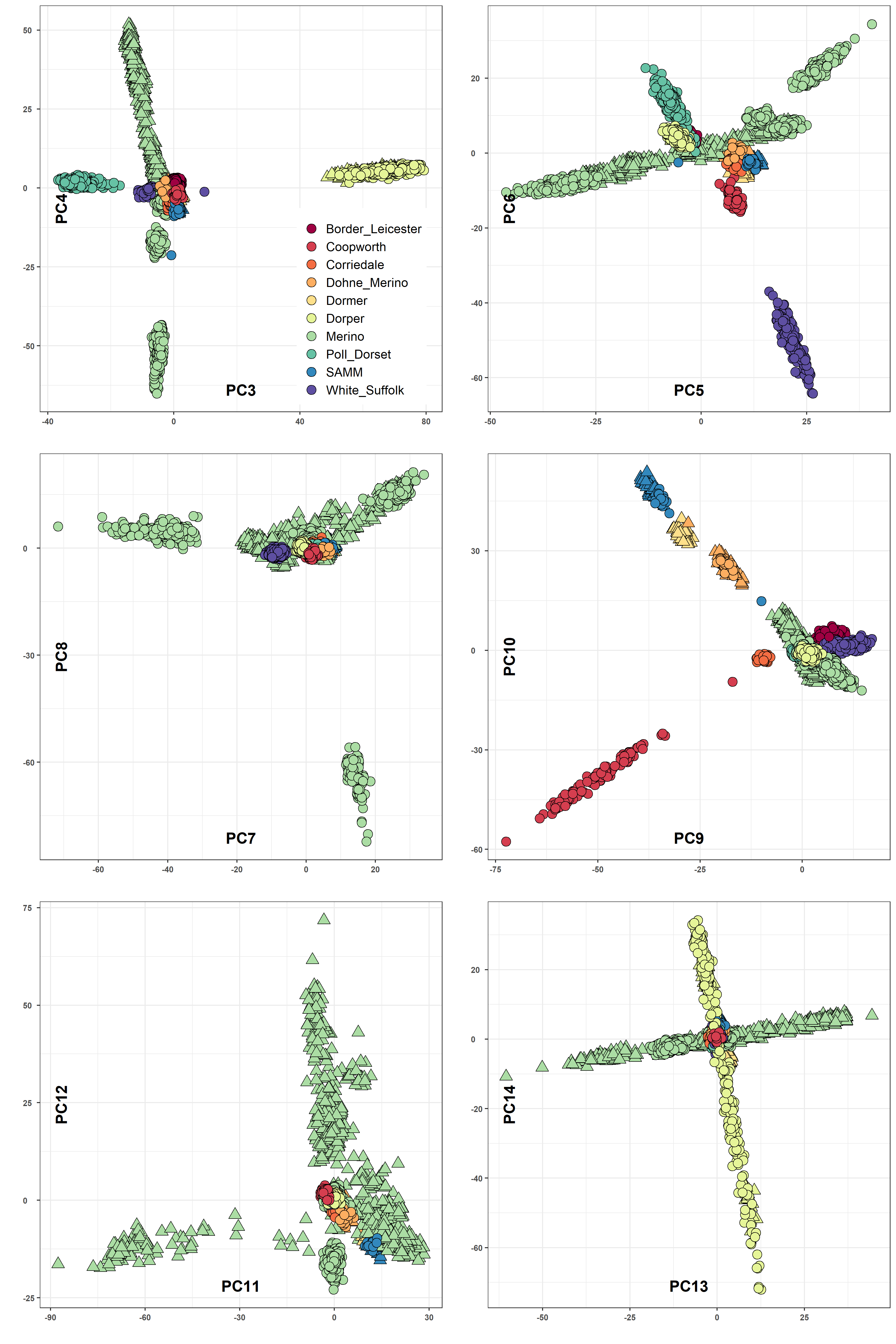

Supplement: Supplementary file 2 — Additional file 2: Fig. S2. The third (PC3) to fourteenth (PC14) principal components of genotypes identified by breed group. [file 12864_2021_8020_MOESM2_ESM.png]

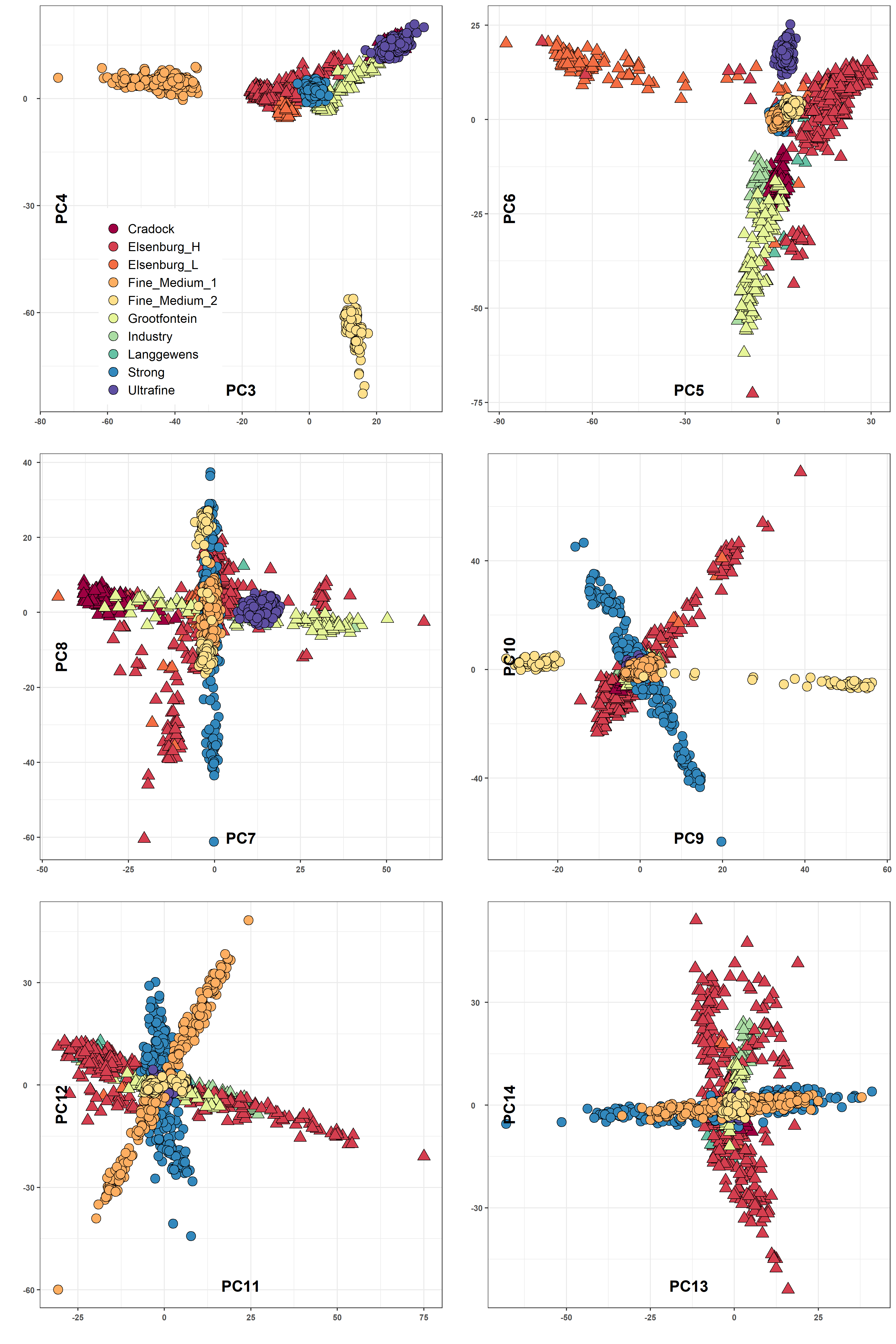

Supplement: Supplementary file 3 — Additional file 3: Fig. S3. The third (PC3) to fourteenth (PC14) principal components of genotypes identified by bloodline. [file 12864_2021_8020_MOESM3_ESM.png]

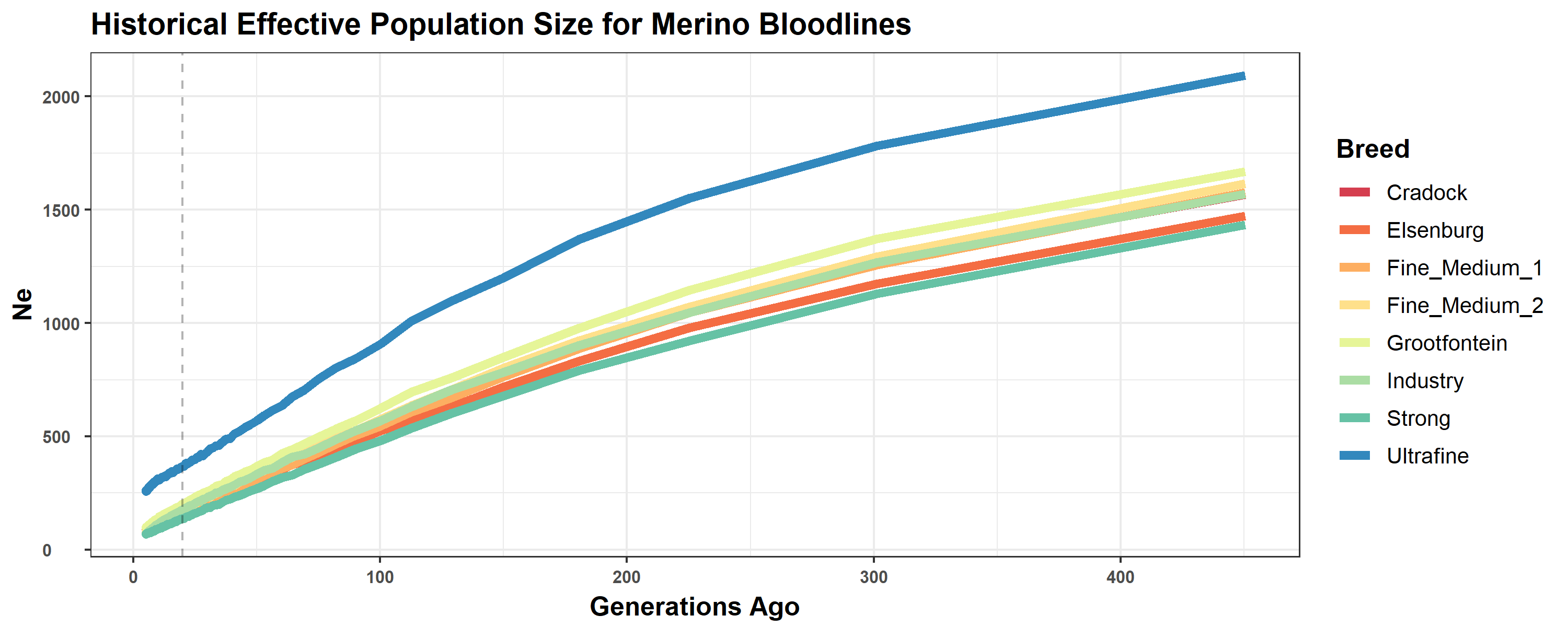

Supplement: Supplementary file 4 — Additional file 4: Fig. S4. Historical effective population sizes (Ne) for Merino bloodline groups from 2 to 400 generations ago. The vertical-line represents the timepoint used for ‘current’ Ne [file 12864_2021_8020_MOESM4_ESM.png]

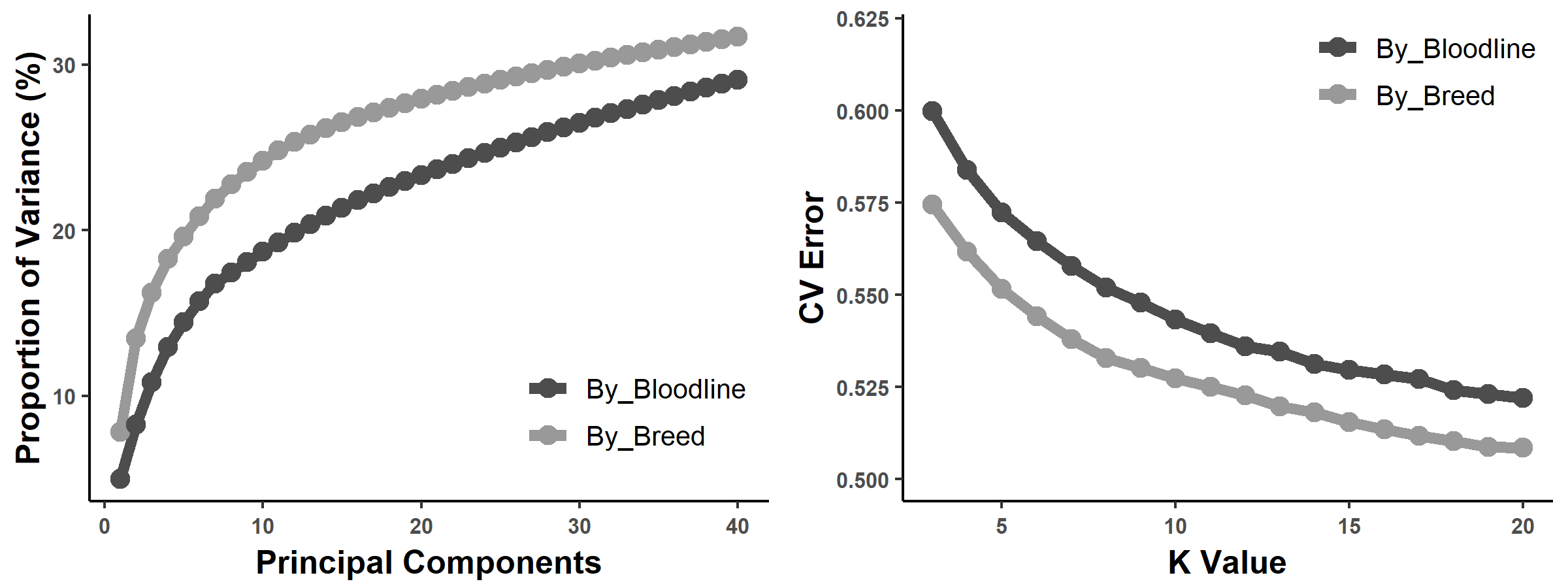

Supplement: Supplementary file 5 — Additional file 5: Fig. S5. (A) Cumulative proportions of variance (POV) explained by the first 40 principal components of 'by breed' and 'by Merino bloodline' analysis. (B) The "CV-error" statistic resulting from sequential ADMIXTURE runs or K = 3 to K = 20 of 'by breed' and 'by bloodline' analysis. [file 12864_2021_8020_MOESM5_ESM.png]
